# Discovery and comparative genomic analysis of elk circovirus (ElkCV), a novel circovirus species and the first reported from a cervid host

**DOI:** 10.1038/s41598-020-75577-6

**Published:** 2020-11-11

**Authors:** Mathew Fisher, Thomas M. R. Harrison, Michelle Nebroski, Peter Kruczkiewicz, Jamie L. Rothenburger, Aruna Ambagala, Bryan Macbeth, Oliver Lung

**Affiliations:** 1 National Centre for Foreign Animal Disease, Canadian Food Inspection Agency, Winnipeg, MB Canada; 2 Department of Ecosystem and Public Health and Canadian Wildlife Health Cooperative (Alberta Region), Faculty of Veterinary Medicine, University of Calgary, Calgary, AB Canada; 3 Parks Canada Agency, Banff National Park, Banff, AB Canada; 4 Department of Biological Sciences, University of Manitoba, Winnipeg, MB Canada

**Keywords:** Biological techniques, Computational biology and bioinformatics, Microbiology, Molecular biology

## Abstract

The complete genome sequence of a novel circovirus (elk circovirus (ElkCV) Banff/2019) was determined via high throughput sequencing of liver tissue from a euthanized Rocky Mountain elk (*Cervus canadensis nelsoni*) from Alberta, Canada. The genome is circular and 1,787 nucleotides long, with two major ORFs encoding predicted proteins. Comparative genomic analysis to 4,164 publicly available complete and near complete circovirus genomes showed that ElkCV shares approximately 65% pairwise genome-wide nucleotide identity with the most closely related circovirus species, porcine circoviruses (PCV) 1 and 2 and bat-associated circovirus (BatACV) 11. ElkCV features a stem-loop within the origin of replication region characteristic of circoviruses. However, it differs from those found in PCV1, PCV2 and BatACV11 since it has a longer stem and contains hexamer repeats that overlap the stem in opposing orientations. Interestingly, stem-loop structures of similar length featuring repeats in a similar position and orientation are also seen in some avian circoviruses. Based on the demarcation threshold established by the International Committee on Taxonomy of Viruses (ICTV) for members of *Circoviridae* (80% pairwise genome-wide nucleotide identity), ElkCV represents a novel species and is the first complete circovirus genome reported from a cervid host.

## Introduction

A large number of viruses with highly diverse ssDNA genomes have been discovered through the metagenomic analysis of animal and environmental samples. The family *Circoviridae* consists of viruses with covalently closed, single stranded DNA genomes and are the smallest autonomously replicating animal pathogens known^1^. Members of this family have been discovered in a number of mammals and birds and can contribute to economically-significant disease in pigs (e.g. PCV2) and birds (e.g. bird and feather disease virus (BFDV))^1^. Infection with other circoviruses appear to be subclinical, but may be immunosuppressive and increase disease severity with co-infecting pathogens^2–6^. The two current genera within the family *Circoviridae*, *Circovirus* and *Cyclovirus*, are distinguished by two elements: the length of the intergenic region and the position of the origin of replication in relation to the coding regions^7^. The demarcation threshold for viral species within these genera, as established by the International Committee on Taxonomy of Viruses (ICTV), is 80% genome-wide nucleotide pairwise identity^7^.

Circoviruses are non-enveloped viruses with genomes ranging from approximately 1.8–2.1 kb in size. Their genomes have an ambisense organization and contain two major ORFs greater than 600 nucleotides on opposing strands of the dsDNA replicative form^8^. The two major ORFs encode the replication-associated protein (Rep) on the virion strand and the capsid protein (Cp) on the complementary strand. Circoviruses have also been reported to encode additional proteins. For example, porcine circoviruses encode more than 6 proteins and both porcine circovirus 1 (PCV1) and porcine circovirus 2 (PCV2) encode VP3 and ORF4 which have apoptotic and potential anti-apoptotic activity, respectively^1^. A number of genomic features characteristic of circoviruses have been described including an origin of replication region containing hexamer repeats and a presumed stem-loop structure with a highly conserved nonamer sequence at the apex that is flanked by inverted-repeat (palindromic) sequences^9^, conserved amino acid motifs within Rep^1^, and a Cp with an N-terminus rich in arginine or other basic amino acids^1^.

## Materials and methods

### Case history

On July 17, 2019, a yearling male Rocky Mountain elk (*Cervus canadensis nelsoni*) was observed in sternal recumbency in Banff National Park, Canada. The elk displayed labored breathing and was unable to stand when prompted. In addition, blood was noted leaking from the sheath and the rectum which also contained formed feces covered in a thick mucoid layer. It was humanely euthanized and the carcass was frozen and subsequently submitted to the Alberta Region of the Canadian Wildlife Health Cooperative at the University of Calgary Faculty of Veterinary Medicine for diagnostic investigation. Lesions included multisystemic hemorrhage, splenomegaly and pulmonary edema, which prompted the submission of tissues to the Animal Health Laboratory, Guelph, Ontario for PCR assays to detect epizootic hemorrhagic disease virus (EHDV) and bluetongue virus (BTV) with negative results. A subsequent PCR assay for *Babesia odocoilei* at the Vector Borne Disease Diagnostic Lab, North Carolina State University was also negative. No live animals were handled for sample collection in the current study.

Five tissues (kidney, lung, liver, spleen and lymph node) were sent to the Canadian Food Inspection Agency’s (CFIA) National Centre for Foreign Animal Disease (NCFAD), Winnipeg, Manitoba for confirmatory testing by PCR for BTV and EHDV. All tissues were negative by established diagnostic PCR assays and therefore submitted for further characterization via high-throughput sequencing (HTS).

### Sample processing and high-throughput sequencing

HTS was performed on an Illumina MiSeq instrument using a previously reported viral enrichment method^10,11^. HTS libraries were prepared from submitted tissues and a water negative control in parallel. Nucleic acid extraction was performed using the Ambion MagMax Viral RNA Isolation Kit (Thermo Fisher Scientific) and eluted in UltraPure Water (Sigma). In order to allow broad metagenomic detection of viruses with either DNA or RNA genomes, reverse transcription was performed on extracted nucleic acid using the Invitrogen SuperScript IV First-Strand Synthesis System (SSIV) (Thermo Fisher Scientific) according to the manufacturer’s instructions with the addition of a tagged random nonamer primer (GTTTCCCAGTCACGATANNNNNNNNN). Sequenase Version 2.0 DNA Polymerase (Thermo Fisher Scientific) was used to perform second strand synthesis. Sequence-independent single-primer amplification (SISPA) was performed using AccuPrime Taq DNA Polymerase System (Thermo Fisher Scientific) and the manufacturer’s recommended conditions. Here, cDNA was universally amplified using a primer complementary to the tag introduced during reverse transcription. The SISPA product was purified using Genomic DNA Clean & Concentrator-10 columns (Zymo Research), quantified using the Qubit dsDNA HS Assay Kit on the Qubit 3.0 Fluorometer (Thermo Fisher Scientific) and subsequently used for HTS library preparation.

Sequence library preparation and enrichment were performed using the KAPA HyperPlus library preparation kit (Roche Diagnostics) and a custom pan-vertebrate virus targeted enrichment probe panel^12^ according to the Nimblegen SeqCap EZ HyperCap Workflow User’s Guide (Roche) and the manufacturer’s recommendations. Enriched libraries were quantified, pooled and sequenced on a V2 flowcell using a 500 cycle kit (Illumina).

### Genome assembly and analysis

**Figure 1:**
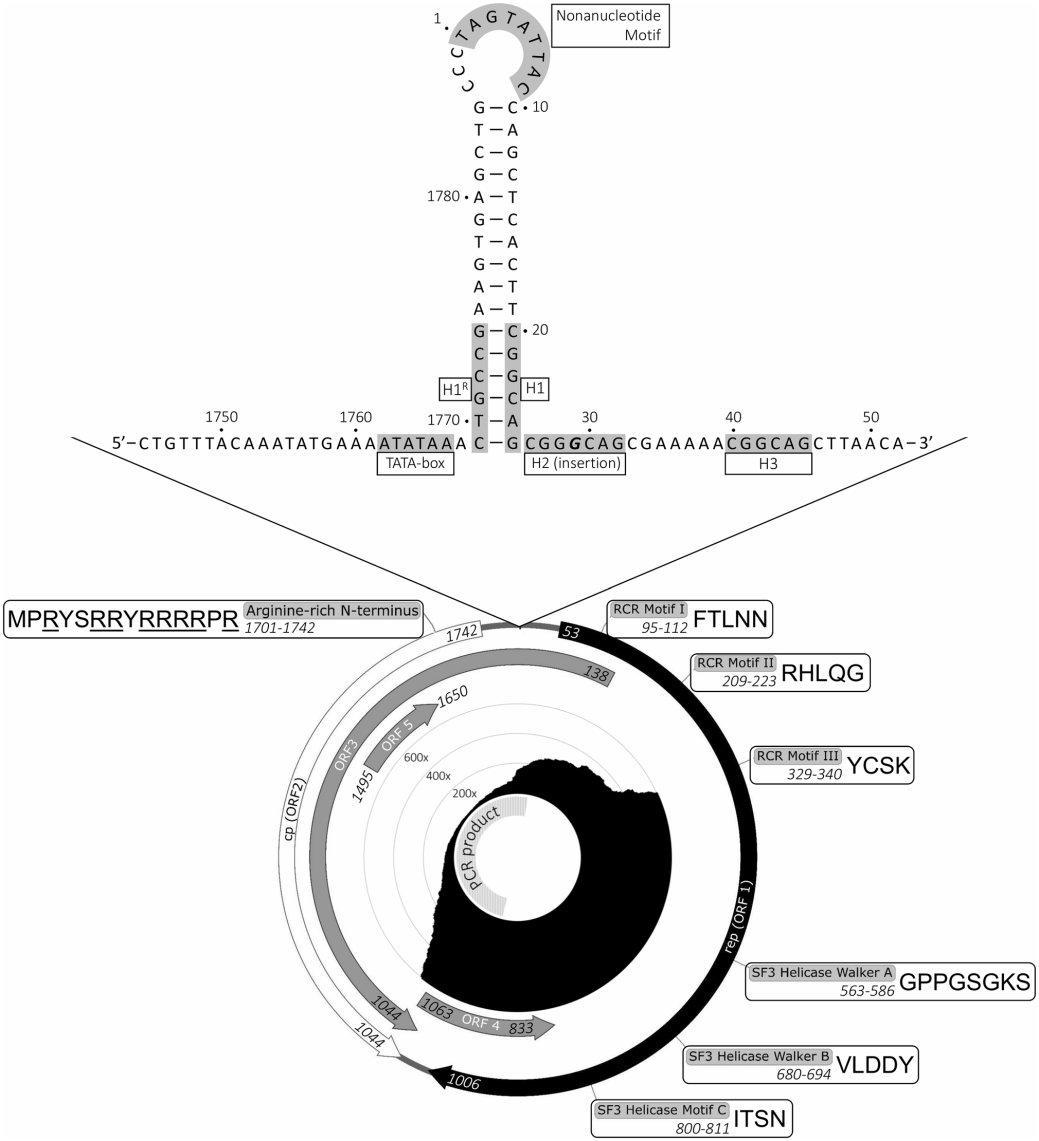
Structure of the elk circovirus Banff/2019 genome. Bottom: Complete circular genome map and coverage plot. On the outside, start and end nucleotide position of ORFs and motifs are indicated in italics. Amino acid sequences (5′–3′) are shown in single letter code beside motif names and positions. ORFs were visualized using SnapGene Viewer v5.0.7 (snapgene.com). Inside the annotated circular genome is a circular plot (generated using GView v1.7^38^) showing coverage at each nucleotide position based on reference mapping of the liver-derived metagenomic sequencing data to the assembled genome. Inside the coverage plot, an 874 nt PCR product (from nucleotide positon 963 to 49) that was subsequently sequenced in order to confirm a region with low relative coverage, is indicated (coverage data not shown). Top: Expanded view of the origin of replication stem-loop region. Nucleotide position and features of note are labelled. Hexamer repeats are labelled as H1-3 with a reverse orientation repeat on the opposite stem of the stem-loop compared to H1 labelled as H1^R^. A single base insertion located in the H2 hexamer repeat sequence that is also found in BatACV11 is indicated in bold and italics.

Initial exploratory metagenomic analysis was carried out using the in-house nf-villumina Nextflow workflow (v2.0.0)^13^. First, nf-villumina removed Illumina PhiX Sequencing Control V3 reads using BBDuk^14^, followed by adaptor removal and quality filtering using fastp^15^. Subsequently, filtered reads were taxonomically classified using Centrifuge^16^ and Kraken2^17^ with an NCBI nt Centrifuge index built February 14, 2020 and Kraken2 index of NCBI RefSeq sequences of archaea, bacteria, viral and the human genome GRCh38 downloaded and built on March 22, 2019. Reads categorized as either viral or unclassified were retained for de novo assembly by both Unicyler^18^ and Shovill^19^. Contigs with homology to circoviruses were observed in the liver and kidney based on the output of both assemblers following a blastn (v2.9.0)^20^ query (default parameters except “-evalue 1e-6”) of the nr/nt database (downloaded January 9, 2020). The potential circovirus contigs from both assemblers and tissues were compared and found to be identical, with the exception of the extreme ends which were manually trimmed using Geneious Prime v2019.1.3^21^. The resulting trimmed sequence was used in an iterative reference assembly (using default settings and 5 iterations) with the filtered viral and unclassified reads using Geneious Prime v2019.1.3^21^, which resulted in a majority consensus sequence longer than the trimmed reference sequence. The resulting majority consensus sequence was manually inspected for an overlap region, which would be expected in a linearized complete circular genome. After an overlap region was identified, the consensus sequence was manually edited to produce a complete linear genome which was then circularized and used in a final reference mapped assembly with the unfiltered, adapter trimmed reads using Geneious Prime v2019.1.3^21^. Visualization of the genome was done using SnapGene Viewer v5.0.7 (snapgene.com) for mapping of open reading frames and GView v1.7^22^ for generation of a coverage plot (Fig. 1). The complete genome is available on NCBI under accession MN585201.

In order to confirm the sequence of a region with low coverage relative to the rest of the genome, primers were designed (Forward: TTTCCCCACCCTGCAATGAG, Reverse: TAAGCTGCCGTTTTTCGCTG) to high coverage flanking regions to produce an 874 nt amplicon from nucleotide position 963 until 49 (Fig. 1). Extracted liver and kidney samples were amplified using the designed primers and Q5 High-Fidelity 2X Master Mix (New England Biolabs) according to manufacturer’s recommendations. Thermal cycling was carried out at 98 °C for 30 s followed by 35 cycles of 98 °C for 10 s, 67 °C for 30 s and 72 °C for 1 min. Cycling was followed by a final extension at 72 °C for 2 min. The amplified product was visualized using a QIAxcel (QIAGEN), where bands of the expected size were observed in both samples. PCR products were sequenced with the Nextera XT library preparation kit on a V2 micro flowcell using a 500 cycle kit (Illumina). The resulting reads was mapped to the previously generated complete genome sequence using Geneious Prime v2019.1.3^21^.

Circrotate, an in-house developed tool which automatically linearizes circular genomes after rotation to a predefined starting point, was used to linearize all publicly available circovirus genomes from GenBank (n = 4,222, November 29, 2019) to begin at the highly conserved origin of replication (NAGTATTAC or YATTATTAC). Sequences that could not be processed by the initial Circrotate run (n = 231) were examined individually. Of these sequences, 58 were determined to be incomplete genomes and were discarded, 92 were successfully rotated on a second Circrotate run using an origin of replication consensus sequence with additional degeneracy added (NANTATTAC), and the remaining 81 were rotated to the beginning of the Rep gene. Pairwise alignment between each linearized complete circovirus genome (n = 4,164) and the novel genome was performed using the Needleman-Wunsch algorithm from the EMBOSS Needle tool (v6.6.0.0)^22^ with default settings. These individual pairwise alignments were used to assess the pairwise sequence identity between the novel genome and other circovirus genomes.

**Figure 2:**
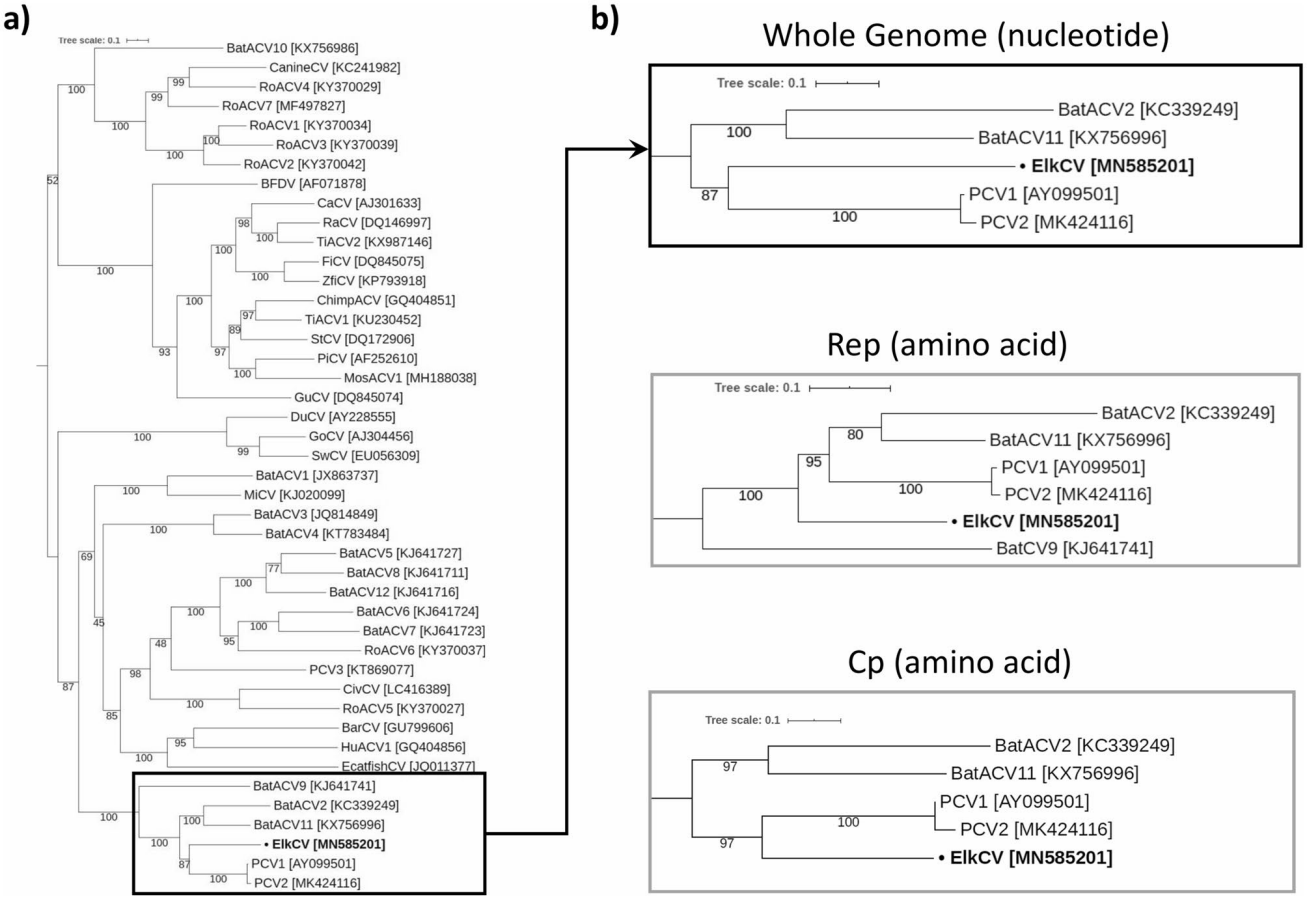
Maximum likelihood phylogenetic trees of representative sequences within the genus *Circovirus*. Sequences were aligned using MAFFT^24^, trees generated using IQ-Tree^23^ on find best model setting with ModelFinder^25^ with 1000 ultrafast bootstraps^26^ and visualized with iTOL^27^. ElkCV is indicated in bold text and with “•” before the sequence name in all trees. (**a**) Full mid-point rooted phylogenetic tree generated from representative *Circovirus* complete genome nucleotide sequences (model TIM + F + R5). (**b**) Pruned trees showing only ElkCV and sequences on closely related branches from complete genome nucleotide sequences (top black box), Rep protein amino acid sequences (middle gray box, model LG + I + G4) and Cp protein amino acid sequences (bottom gray box, model PMB + R3).

Maximum-likelihood phylogenetic trees were generated with IQ-TREE^23^ from MAFFT^24^ multiple sequence alignments (MSA) of the novel genome and other representative *Circovirus* complete genome nucleotide sequences, Rep amino acid and Cp amino acid sequences. IQ-Tree phylogenetic trees were produced using the substitution models indicated in Fig. 2, as selected by ModelFinder^25^, with 1,000 ultrafast bootstraps^26^ and visualized using Interactive Tree Of Life (iTOL)^27^.

## Results and discussion

High throughput sequencing was performed directly on nucleic acid extracted from five different tissue sample types in order to reduce the chance of contamination from environmental or other sources. Sequencing performed on the samples resulted in the assembly of a complete circular genome of a novel circovirus species (elk circovirus strain Banff/2019) from the liver and a partial genome from the kidney. Based on a reference assembly using metagenomic sequencing data, 29,812 reads (0.81% of total reads) from the liver and 813 reads (0.03% of total reads) from the kidney matched the novel circovirus genome. Metagenomic sequencing reads from the liver derived material mapped across 100% of the novel genome with a mean coverage of 4573 × and a minimum coverage of 4 ×. Metagenomic sequencing reads from the kidney derived material mapped across 73.2% of the novel genome with a mean coverage of 105 ×. Majority consensus sequences were generated from the reference assemblies of both tissues (where coverage was > 2 ×) and showed 100% nucleotide identity to each other.

Subsequently, amplicon sequencing was performed on an 874 nt PCR product covering nucleotide position 963 to 49 of the genome, which had lower relative coverage. Based on reference assemblies using amplicon sequencing data, 724,378 reads (97.30% of total reads) from the liver and 717,046 reads (98.46% of total reads) from the kidney mapped to the previously assembled novel circovirus genome sequence. The resulting majority consensus sequences from the liver and kidney had 100% nucleotide identity to each other and to the overlapping region of the previously assembled novel circovirus genome sequence. After combining the metagenomic and amplicon sequencing data and repeating reference assembly, the minimum coverage for the liver derived material increased to 208 ×.

The elk circovirus (ElkCV) genome has two major ORFs with homology to reported circovirus proteins. ORF1 is located on the virion strand and encodes a predicted 317 aa replication-associated protein (Rep) which contains several amino acid motifs conserved in circovirus genomes (Fig. 1). ORF2 is on the complementary strand and encodes a predicted 232 aa capsid protein (Cp) that contains an arginine-rich N-terminal region characteristic of circoviruses. The genome contains an additional 3 ORFs (ORFs 3–5) encoding putative proteins that do not show substantial similarity to other known proteins based on blastn^20^ and blastx^20^ queries of the NCBI nr/nt database and HMMer3 hmmsearch^28^ against the Pfam HMM DB (v33.1)^29^ (performed June 26, 2020). ORF3 partially overlaps with ORF2 and is in the same reading frame so it displays similarity to other circovirus Cp proteins in overlapping regions. However, in regions that do not overlap with ORF2, ORF3 did not show similarity to other proteins. ORF3 and ORF4 are on the complementary strand and potentially encode putative 293 and 76 aa proteins, respectively, while ORF5 is on the virion strand and potentially encodes a putative 51 aa protein.

EMBOSS Needle (v6.6.0.0)^22^ was used to sequentially perform pairwise alignment of the ElkCV genome to all Circrotate linearized genomes. It was determined that the closest match was a PCV1 sequence (CT-PCV-P7 accession number AY099501.1) with a pairwise genome-wide nucleotide identity of 65.7% (Table 1). The viral species with the highest similarities were PCV1 with 63.6–65.7% nucleotide identity and PCV2 with 56.3–64.7% nucleotide identity. These results are consistent with phylogenetic analysis which showed that ElkCV clusters with PCV1 and 2 as well as several species of bat-associated circovirus (BatACV) (Fig. 2). The percent identity of ElkCV to all other reported sequences falls below the 80% identity cut-off established by the ICTV for species demarcation in the family *Circoviridae*, making it a novel species within the genus *Circovirus*.

**Table 1:**
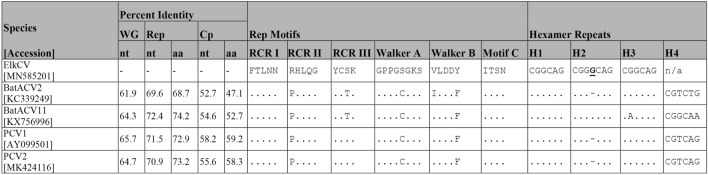
Comparison of the elk circovirus Banff/2019 genome to the most closely related circovirus genomes currently known.

Percent identity is shown between the elk circovirus genome and the genomes of closely related species based on nucleotide alignments of the whole genome (WG) as well as nucleotide (nt) and amino acid (aa) alignments of the Rep and Cp genes. The sequences of the conserved motifs found within Rep and hexamer repeats found near the origin of replication are shown. Nucleotide or amino acid resides identical to elk circovirus are indicated with a period and any differences are shown. A single base insertion in the H2 repeat of both elk circovirus and BatACV11 is bolded and underlined, while the gap in other sequences where this base is absent are indicated with “–”.

ElkCV has a number of features reported in other circovirus genomes including a nonanucleotide motif (TAGTATTAC) that forms the origin of replication at the apex of a stem-loop which is located within a 97 nt intergenic region (Fig. 1). This loop has a stem length of 16 nt, making it similar in length to those seen in some avian circoviruses^30^. Interestingly, this is longer than the 11 nt stem seen in both PCV1 and 2, which are the most similar sequences to ElkCV based on both phylogenetic analysis and percent identity across the whole genome, Rep and Cp. This intergenic region also features a putative TATA-box upstream of the stem-loop structure and several hexamer repeat sequences.

Circoviruses have nucleotide repeats (named H1–H4) that can neighbour or in some cases be within the stem-loop and can vary in number, sequence, position and orientation based on species. PCV1 and 2 typically have four sequential repeats of the hexamer CGGCAG, referred to as H1-H4, which are organized into two tandem repeats located beside the stem-loop^31^. The ElkCV genome has an identical H1 and H3, but an H2 with a single nucleotide insertion (CGG**G**CAG) and is missing H4. An identical H2 with the same insertion is present in BatACV11 (accession KX756996.1) as well as two unclassified BatACV sequences which appear to be type 11 (accessions MH760364.1 and MK070856.1). Due to this insertion, the entire H1/H2 tandem repeat of these BatACV sequences is identical to ElkCV. While ElkCV appears to lack H4 (which is present in the aforementioned PCV and BatACV genomes), the H1 repeat is located within the stem of the stem-loop and therefore it has a repeat within the same intergenic region on the opposite side of the stem in reverse orientation (H1^R^) (Fig. 1, top). While these H1 and H1^R^ repeats within the stem-loop structure do not appear in PCV1, PCV2 or BatACV11, some avian circoviruses have repeats in a similar position and orientation on the stem-loop. Raven circovirus (accession DQ146997.1), starling circovirus (accession DQ172906.1) and finch circovirus (accession DQ845075.1) feature stem-loops of 19, 17 and 15 nt, respectively, with a similar H1 repeat at the base of the stem-loop and an H1^R^ repeat in the opposing direction on the opposite stem^32^. This is consistent with our observation that the length of the stem-loop of ElkCV is much more similar to those found in some avian circoviruses. Although the position and orientation of these repeats on the stem-loop are similar to those in ElkCV, it is worth noting that the length and sequence of the repeat itself is quite different. The presence of these features is further supported by previous studies of PCV1 which showed that if the H1/H2 tandem is present then H3/H4 is non-essential^33,34^ and that a PCV1 mutant was still viable with an H1^R^ insertion on the left stem of the stem-loop which generated a longer stem with overlapping H1 repeat^35^.

Analysis of the ORF encoding Rep (ORF1) shows the presence of three motifs involved in rolling circle replication (RCR Motif I, II and III), and three superfamily 3 (SF3) helicase motifs (Walker A, B and Motif C) (Fig. 1 and Table 1). Amino acid residues neighbouring some of these motifs match proposed DNA binding specificity determinants specific to PCV1 and PCV2^31^. Comparison of the predicted ElkCV Cp protein (ORF2) with those from other circoviruses shows the presence of a characteristic N-terminal region rich in arginine or other basic amino acids (MP**R**YS**RR**Y**RRRR**P**R**).

The relationship between ElkCV and the lesions in this case remains unclear. In dogs, there has been a proposed association between hemorrhagic gastroenteritis with vasculitis and canine circovirus^36^; however, a case–control study also identified the virus in healthy control dogs, suggesting that infection is widespread^37^. Similarly, PCV1 is not known to cause disease in pigs and many pigs infected with PCV2 do not develop severe lesions. There is also an indication that infection with circoviruses tends to lead to more severe outcomes in instances of co-infection^2–6^. Analysis of additional cases would be useful to establish a potential association between the presence of the virus and clinical disease. The PCR that was designed and successfully generated an 874 nt amplicon from the extracted liver and kidney samples could potentially be used for future screening of elk circovirus when additional cases becomes available.

## Data Availability

The complete genome is available on NCBI under accession MN585201.
